# Conformational analysis and intramolecular interactions in monosubstituted phenylboranes and phenylboronic acids

**DOI:** 10.3762/bjoc.9.125

**Published:** 2013-06-11

**Authors:** Josué M Silla, Rodrigo A Cormanich, Roberto Rittner, Matheus P Freitas

**Affiliations:** 1 Department of Chemistry, Federal University of Lavras, P.O. Box 3037, 37200-000, Lavras, MG, Brazil; 2 Chemistry Institute, State University of Campinas, P.O. Box 6154, 13083-970, Campinas, SP, Brazil

**Keywords:** conformational analysis, hydrogen bond, interactions with boron, monosubstituted phenylboranes, phenylboronic acids

## Abstract

A ^1^*^TS^J*_F,H(O)_ coupling pathway, dictated by a hydrogen bond, in some 2-fluorobenzoic acids has been observed, while such an interaction does not occur in 2-fluorophenol. Thus, this work reports the conformational analysis of 2-fluorophenylboronic acid (**1**), in order to evaluate a possible intramolecular OH∙∙∙F hydrogen bond in comparison to an n_F_→p_B_ interaction, which mimics the quantum n_F_→σ*_OH_ hydrogen bond that would be expected in 2-fluorophenol. 2-Fluorophenylborane (**3**), which does not experience hydrogen bonding, was used to verify whether n_F_→p_B_ interaction governs the conformational equilibrium in **1** due to a predominant OH∙∙∙F hydrogen bond or to other effects. A series of 2-X-phenylboranes (X = Cl, Br, NH_2_, PH_2_, OH and SH) were further computationally analyzed to search for electron donors to boron, capable of influencing the conformational equilibrium. Overall, the intramolecular OH∙∙∙F hydrogen bond in **1** is quite stabilizing and dictates the ^1^*^h^J*_F,H(O)_ coupling constant. Moreover, electron donation to the empty p orbital of boron (for noncoplanar BH_2_ moiety relative to the phenyl ring) is also significantly stabilizing for the NH_2_ and PH_2_ derivatives, but not enough to make the corresponding conformers appreciably populated, because of steric effects and the loss of π_CC_→p_B_ resonance. Thus, the results found earlier for 2-fluorophenol about the lack of intramolecular hydrogen bonding are now corroborated.

## Introduction

Boronic acid derivatives have been widely studied because of their good performance as pharmaceutical agents, serving in the development of enzyme inhibitors of peptidases/proteases, proteasomes, arginase, nitric oxide synthase (NOS), and transpeptidases [1–2]. Other important studies incorporate the boronic acid moiety into amino acids and nucleosides as antitumor and antiviral agents [3–4]. Indeed, the great importance of aromatic boronic acids to biological and pharmaceutical purposes has been reported, as well as the interest to introduce a boronic acid moiety in organic molecules [5]. Boron has been shown to bind with nitrogen in order to form a ring in 2-(*N*,*N*-dimethylaminomethyl)phenylboronic acid [6], thus reflecting its electron acceptor ability through intramolecular interactions. In addition, computational studies have been performed to evaluate the difference in affinity of boron towards oxygen and nitrogen electron pairs in 2-aminocarbonylphenylboronic acid (2-AC-PBA) and its corresponding ester, ethanediol(2-aminocarbonyl)phenylboronate (ED-2-AC-PB), that has been identified for some conformer interactions of type B–N and B–O, in addition to typical intramolecular hydrogen bonds [7]. Niedenzu [8] presented studies in organic synthesis with evidence of intramolecular interactions between boron and electronegative atoms such as F, Cl, O, N and S.

Indeed, boron-containing compounds are Lewis acids, because of the empty p orbital in trivalent boron derivatives. This can be useful to mimic vacant orbitals, which are capable of accepting electrons from symmetry-allowed electron donors, such as the σ*_OH_ orbital as an electron acceptor in hydrogen bonding. For example, 4-bromo-2-fluorophenol is supposed to form intramolecular OH∙∙∙F hydrogen bonds as the governing interaction of the conformational equilibrium and, consequently, of the observed *^1h^J*_F,H(O)_ coupling constant [9]. However, it has been recently found that such coupling in this compound and in 2-fluorophenol itself is better described as ^1^*^TS^J*_F,H(O)_, because of a coupling pathway based on the overlap of proximate electronic clouds rather than hydrogen bonding [10]. Indeed, dipolar effects have been invoked as the determining role of the conformational equilibrium in 2-fluorophenols instead of intramolecular hydrogen bonding [11], contrary to that found elsewhere for 2-monohalogen substituted phenols [12]. In fact, organic fluorine has been found to hardly ever participate in hydrogen bonding [13], despite the appearance of this interaction in 8-fluoro-4-methyl-1-naphthol [14], 2'-fluoroflavonols [15], 2-fluorobicyclo[2.2.1]heptan-7-ols [16] and 2-fluorobenzoic acids [17]. Recently, OH∙∙∙F hydrogen bonds were found to be difficult to operate in monocyclic compounds when forming five-membered rings, because of geometric restrictions imposed by the rigid rings [18].

**Figure 1:**
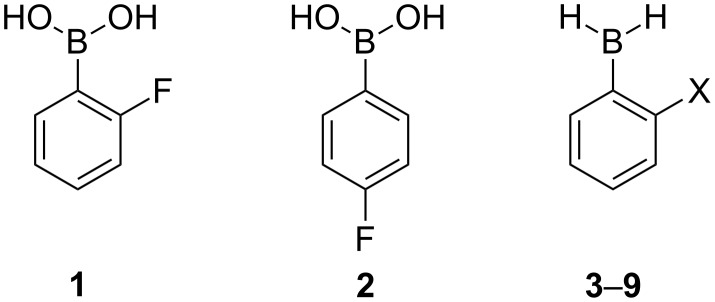
2- and 4-fluorophenylboronic acids (**1** and **2**) and 2-substituted phenylboranes [X = F (**3**), Cl (**4**), Br (**5**), OH (**6**), SH (**7**), NH_2_ (**8**) and PH_2_ (**9**)].

In the present work, 2- and 4-fluorophenylboronic acids were analyzed by using theoretical and spectroscopic tools to account for possible n_F_→*p*_B_ interactions in the ortho isomer, which is similar to the n_F_→σ*_OH_ interaction (charge-transfer contribution for the hydrogen bond) in 2-fluorophenol. Since the OH group in 2-fluorophenylboronic acid is anticipated to participate in intramolecular hydrogen bonding, 2-X-phenylboranes (X = F, Cl, Br, NH_2_, PH_2_, OH and SH) were also evaluated theoretically (Figure 1), in order to account for the importance of n_X_→p_B_ interactions free from interference of the OH∙∙∙X hydrogen bond present in the 2-substituted phenylboronic acids.

In order to achieve these goals, NMR spectroscopy (by means of suitable coupling constants) and theoretical calculations were used. Second-order perturbation analysis of donor–acceptor interactions in the natural bond orbitals (NBO) was used to interpret conformational isomerism in terms of hyperconjugative interactions, in such a way that the molecular interactions are characterized by quantum-mechanical delocalization from filled donor to formally unoccupied acceptor NBOs [19]. In addition to the NBO method, the quantum theory of atoms in molecules (QTAIM) [20] has been widely used to examine the electronic densities leading to possible hydrogen bonds. The QTAIM method describes the electron density (ρ) to define atoms in molecules and their interactions [20–22]. Koch and Popelier [23] established a key criterion to characterize hydrogen bonds in an equilibrium geometry, based on the maximum electron density linking neighboring nuclei, called the bond path (BP). In addition to the BP lines, other parameters are also required to characterize a hydrogen bond, namely the formation of a bond critical point (BCP) for each hydrogen bond, ρ and the ρ Laplacian values (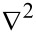
ρ) at the hydrogen bond BCP (ρHBCP and 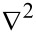
ρHBCP), which lie in the range of 0.002 atomic units (au) to 0.04 au, and 0.024 au to 0.139 au to electrostatic hydrogen bonds, respectively. Moreover, the H atom associated with the hydrogen bond should have a loss of atomic charge [q(H)], an increased atomic energy [*E*(H)], a decreased atomic first dipole moment [*M*_1_(H)] and a decreased atomic volume [*V*(H)] in comparison to those of a H atom not involved in hydrogen bonding [23].

Spectroscopic (Raman, infrared and NMR) and theoretical studies on 2-fluorophenylboronic acid have already been performed previously for assignment purposes, because of the lack of information about this important target for various applications [24]. However, few insights about its conformational isomerism and intramolecular interactions are given, since most of the experimental investigations were devoted to the solid state [24].

## Results and Discussion

**Figure 2:**
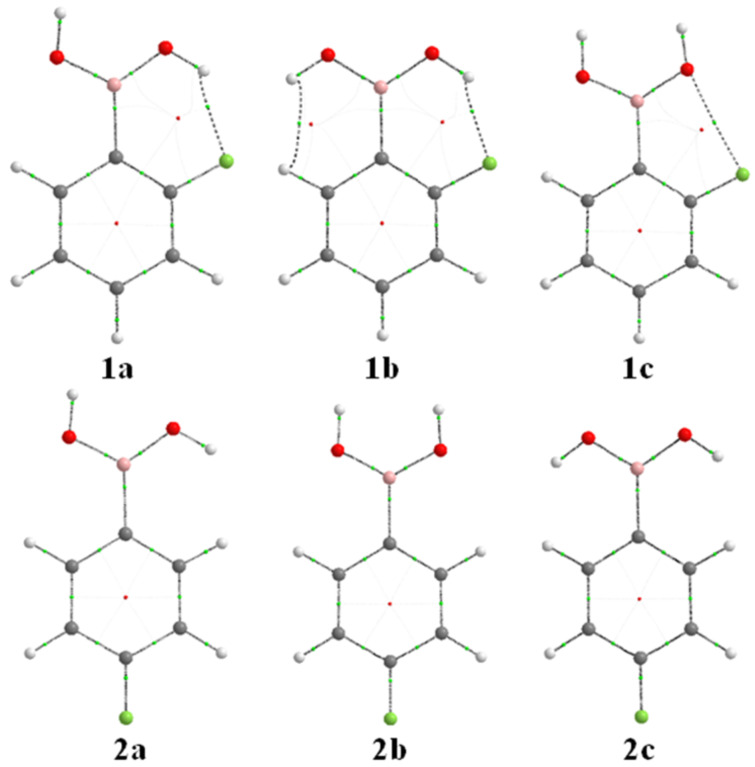
Molecular graphs for the energy minima of 2- and 4-fluorophenylboronic acids. Green dots represent bond critical points and red dots represent ring critical points.

**Figure 3:**
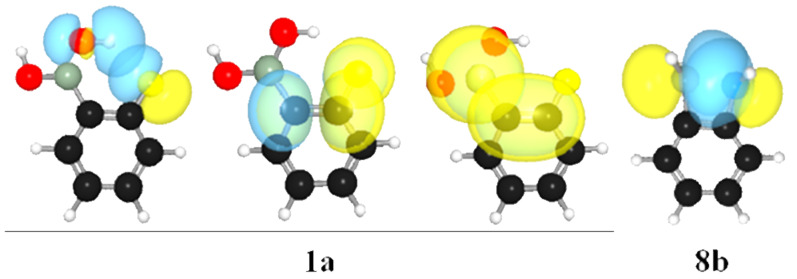
Important hyperconjugative interactions for **1a** (from the left to the right: n_F_→σ*_OH_, n_F_→π*_CC_ and π_CC_→p_B_) and **8b** (n_N_→p_B_) obtained by NBO analysis.

2-Fluorophenylboronic acid undergoes rotational isomerization around the C–B and B–O bonds, giving rise to three energy minima (Figure 2), either in the gas phase or implicit CH_3_CN. Since the energy differences obtained by DFT were similar to those obtained by MP2, the B3LYP/aug-cc-pVDZ level was used for further analysis. The *trans*–*cis* form found elsewhere [24] was characterized here as a saddle point rather than a minimum (an imaginary frequency was found). Conformers **1a** and **1b** exhibit intramolecular hydrogen bonds, which is described in quantum terms as an n_F_→σ*_OH_ interaction (example for **1a** in Figure 3). According to NBO analysis, such an interaction is 3.4 and 3.9 kcal mol^−1^ stabilizing for **1a** and **1b**, respectively, but **1a** is largely dominant both in the gas phase and in solution (Table 1). This is corroborated by the AIM results, whose molecular graphs indicate bond paths between F and H(O) for **1a** and **1b**, in addition to a dihydrogen bond for **1b** due to the reversed polarity of the ring hydrogen (−0.021 au) and the hydroxy hydrogen (+0.588 au), and a nonbonding interaction between F and O in **1c**.

**Table 1:** Conformational energies (in kcal mol^−1^), distances between interacting atoms by hydrogen bond and nonbonding interaction (in Å), and hyperconjugative/repulsive interactions (in kcal mol^−1^).

Parameter	**1a**	**1b**	**1c**	**2a**	**2b**	**2c**

*E*_rel MP2(gas)_^a^	0	3.8	6.3	0	1.6	2.9
*E*_rel DFT(gas)_^a^	0	3.8	5.8	0	1.5	3.2
*E*_rel DFT_^b^	0	3.8	5.8	2.4	3.9	5.6
*E*_rel DFT (CH3CN)_^a^	0	1.6	–	0	0.3	1.4
*E*_rel DFT (CD3CN)_^b^	0	1.6	–	1.3	1.7	2.7
d_O···F_	–	–	2.755	–	–	–
d_OH···F_	2.025	1.984	–	–	–	–
n_F_→σ*_OH_	3.4	3.9	–	–	–	–
n_F_→π*_CC_	16.2	16.5	19.2	18.5	18.3	18.5
π_CC_→p_B_	19.9	18.6	21.8	22.8	25.1	13.2
n_O_→p_B_	304.8	464.3	266.2	255.0	237.2	272.2
Δ*E*_hyper_	2499.4	2667.5	0	80.7	0	2299.7
Δ*E*_Lewis_	2493.1	2665.2	0	79.1	0	2301.4
Total hyperconjugation	25410.7	25578.8	22911.3	24434.7	24354.0	26653.7

^a^Relative energies for the conformers of compounds **1** and **2**, separately. ^b^Relative energies of all structures.

The hyperconjugative energy in each system can be estimated by deleting the electronic transfers from filled to vacant orbitals (antibonding and Rydberg-type orbitals) using the NBO method, and then computing the energy of the resulting system; Lewis-type energy can also be indirectly obtained from this, in such a way that *E*_full_ = *E*_hyperc._ + *E*_Lewis_. Accordingly, **1a** and **1b** were found to be more stabilized due to hyperconjugation than **1c**, which is sterically less hindered (see the lower Lewis-type energy in Table 1); the larger steric and electrostatic repulsion in **1b** is due to the interacting oxygen lone pairs. In polar solvents, the electrostatic effect is minimized and the energy difference between **1a** and **1b** is therefore reduced. The steric term can also be obtained by using the STERIC keyword in NBO, according to the natural energy decomposition analysis scheme, as well as other NBO analysis options [19].

The existence of n_F_→σ*_OH_ electronic delocalization does not guarantee an effective hydrogen bond, since the σ_OH_ is also oriented toward the fluorine substituent, giving rise to a repulsion (4-electron/2-orbital interaction). Thus, AIM calculations were performed to check for the predominant attractive interaction between F and OH rather than a repulsive one. Both **1a** and **1b** conformers show positive 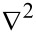
ρ(*r*) values and negative *H*(*r*) values (Table 2), indicating a strong, partially covalent intramolecular hydrogen bond. The remaining criteria established by Koch and Popelier [23] are also satisfied, namely *q*(H), *M*_1_(H), *V*(H) and *E*(H) (Table 3), which were obtained by integration of the atomic basins on the hydrogen participating in the hydrogen bond (**2a** was used as reference because it does not experience hydrogen bonding). The electronic charge [*q*(*H*)] is decreased (more positive), as are *M*_1_(H), *V*(H) and *E*(H) (the latter referred to the destabilization of H after hydrogen bonding) in **1a** and **1b** relative to **2a**. Conformer **1c** was found to be stabilized by a nonbonding F∙∙∙O interaction, which contributes to the formation of pseudo five-membered rings. This would be possible because of an n_F_→π*_CC_ interaction (Figure 3), which contributes to a resonance structure with positive fluorine capable of interacting attractively with oxygen. Table 1 shows that the n_F_→π*_CC_ interaction in **1c** is ca*.* 3 kcal mol^−1^ stronger than in **1a** and **1b**, but not strong enough to make this conformer appreciably populated.

**Table 2:** QTAIM parameters useful to characterize the formation of bonds for **1a–9c**.

Conformers	ρ(*r*)	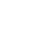 ρ(*r*)	ε	R_BCP_−R_RCP_	*V*(*r*)	*G*(*r*)	*Η*(*r*)

**1a**_OH∙∙∙F_	0.0200	0.0675	0.0374	0.682	−0.0180	+0.0174	−0.0006
**1b**_OH∙∙∙F_	0.0218	0.0746	0.0273	0.700	−0.0197	+0.0192	−0.0005
**1b**_H∙∙∙H_	0.0112	0.0112	0.0424	0.260	−0.0080	+0.0093	+0.0013
**1c**_O∙∙∙F_	0.0105	0.0464	0.3783	0.337	−0.0094	+0.0105	+0.0011
**6a**_H∙∙∙H_	0.0172	0.0487	0.5529	0.454	−0.0108	+0.0115	+0.0007
**7a**_H∙∙∙H_	0.0157	0.0428	0.2596	0.709	−0.0092	+0.0099	+0.0007
**8a**_H∙∙∙H_	0.0124	0.0427	0.6476	0.427	−0.0076	+0.0092	+0.0016
**8b**_ B∙∙∙N_	0.0815	0.1090	0.4231	0.512	−0.1404	+0.0838	−0.0566
**9c**_B∙∙∙P_	0.0663	−0.0071	0.1970	0.629	−0.0595	+0.0288	−0.0307

**Table 3:** Additional atomic properties obtained by QTAIM for hydrogen-bonding atoms (in au).

Conformers	*q*(H)	*M*_1_(H)	*V*(H)	*E*(H)

**2a**_H(OH)_	**+0.592**	**+0.168**	**+21.538**	−**0.3492**
**1a**_OH∙∙∙F_	+0.623	+0.142	+16.655	−0.3356
**1b**_OH∙∙∙F_	+0.621	+0.140	+16.222	−0.3375

**Figure 4:**
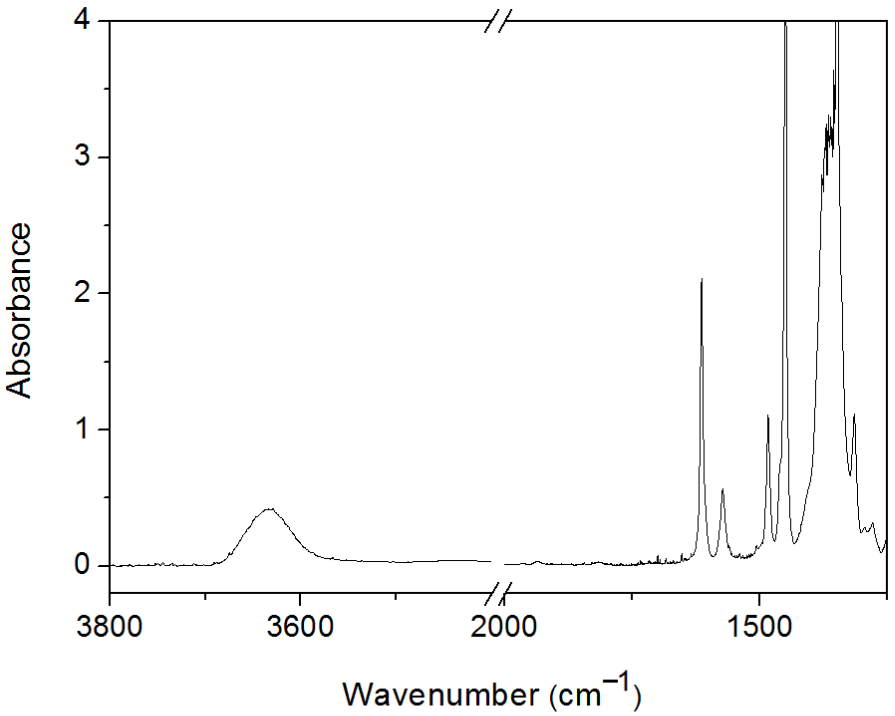
Infrared spectrum of 2-fluorophenylboronic acid in 0.1 M chloroform solution.

Overall, **1a** was calculated to be practically the only existing conformer, because of its high hyperconjugative stabilization compared to **1c** and lower steric repulsion compared to **1b**; also, it is greatly favored by an F∙∙∙HO intramolecular hydrogen bond. This information can be theoretically checked by comparing the geometries of **1** and **2** (where F∙∙∙HO intramolecular hydrogen bonding is not possible): while **2b** is more stable than **2c**, because it prevents the interaction between the oxygen lone pairs, **1b** is more stable than **1c**, even exhibiting such a repulsive interaction, because **1b** allows a F∙∙∙HO intramolecular hydrogen bond. The lack of intermolecular hydrogen bonding and the presence of only one conformer in solution can be readily assessed by analyzing the infrared spectrum of 2-fluorophenylboronic acid (**1**) in 0.1 M CHCl_3_ solution, where a symmetric, high-frequency band (centered at 3635 cm^−1^) associated with the OH stretching mode is observed (Figure 4). In the solid state, where 2-fluorophenylboronic acid is expected to be intermolecularly coordinated, the O–H stretching modes are observed at 3467 cm^−1^ [24].

**Figure 5:**
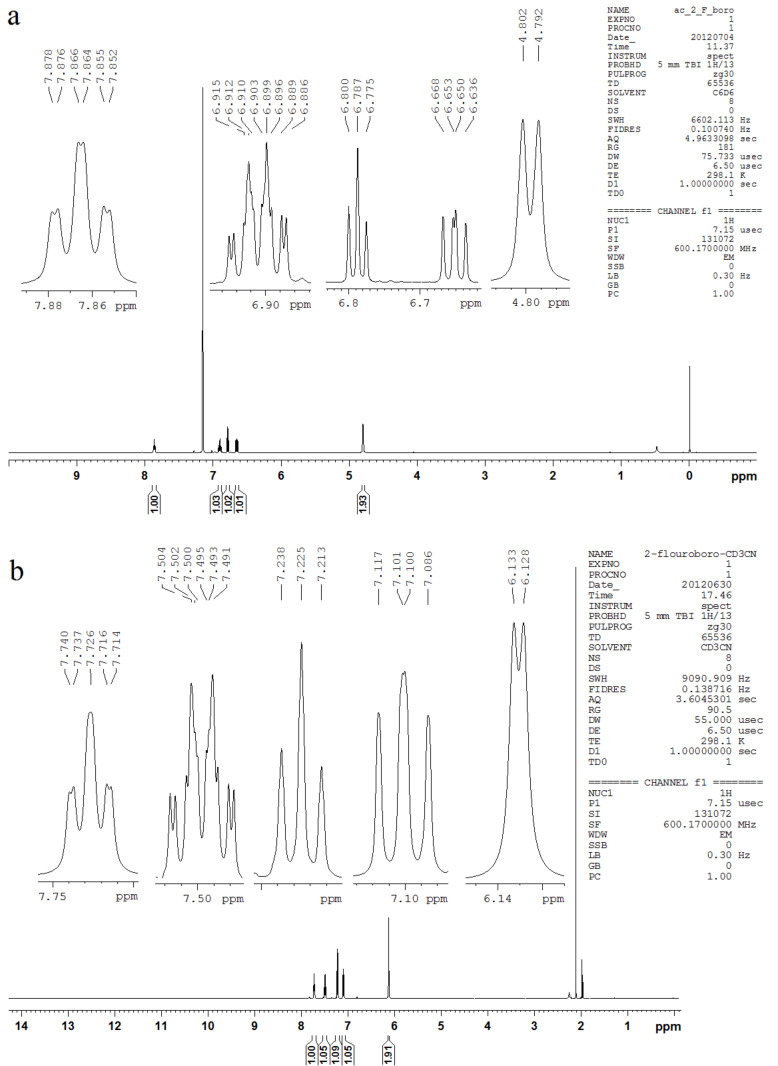
^1^H NMR spectrum for **1** in (a) C_6_D_6_ solution (2 mg mL^−1^) and (b) CD_3_CN solution (20 mg mL^−1^).

**Figure 6:**
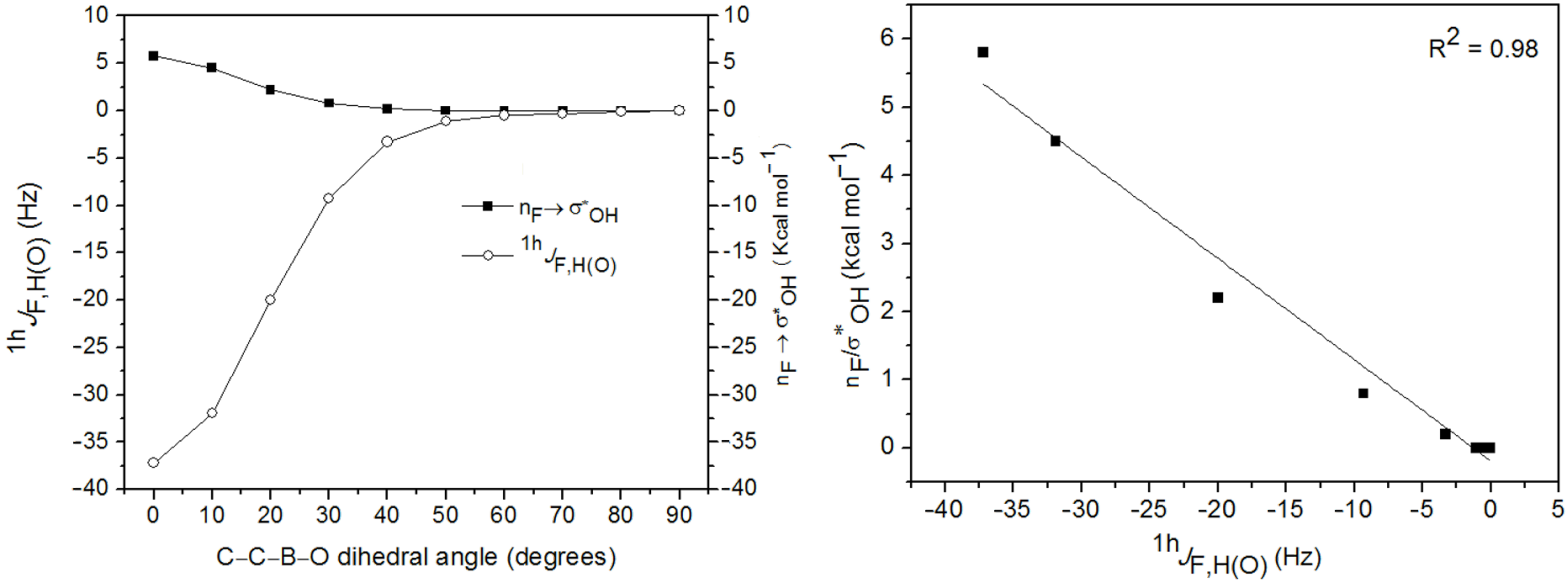
Angular dependence of ^1^*^h^J*_F,H(O)_ and n_F_→σ*_OH_ in **1a**, obtained at the BHandH/EPR-III (*J*) and B3LYP/aug-cc-pVDZ (n_F_→σ*_OH_) levels.

The F∙∙∙HO intramolecular hydrogen bond can be probed by using the ^1^*^h^J*_F,H(O)_ coupling constants. Indeed, the H(O) signal for 2-fluorophenylboronic acid in C_6_D_6_ and CD_3_CN solutions is a doublet with ^1^*^h^J*_F,H(O)_ of 6.0 and 3.0 Hz, respectively (Figure 5). These coupling constants are much lower than those calculated at the BHandH/EPR-III level (−18.9 and −21.9 Hz for **1a** and **1b**, respectively), because of the experimental acidity of these hydrogen atoms, which can be proved by the smaller coupling constant value in CD_3_CN (dissociation enhanced compared to C_6_D_6_). However, there is a high correlation between ^1^*^h^J*_F,H(O)_ and the n_F_→σ*_OH_ interaction in **1a** (Figure 6, R^2^ = 0.98), indicating the possible coupling pathway. The through-hydrogen-bond coupling can be assigned rather, than a pathway based on superposition of electronic clouds (like in 2-fluorophenol [10]), by analyzing the percentage s-character in the fluorine lone pairs (Table 4). Attractive interactions increase the percentage s-character of lone pairs (LP) involved in the transmission mechanism of coupling constants. While LP(1) in **1a** and **1b** exhibits a decreased percentage s-character compared to **1c** (where hydrogen bonding is not possible), the percentage s-character in LP(2) is compensated in **1a** and **1b**, confirming an overall attractive interaction between F and H(O).

**Table 4:** Percentage s-character of fluorine lone pairs in the conformers of 2-fluorophenylboronic acids.

Conformer	LP_F_(1)	LP_F_(2)	LP_F_(3)

**1a**	67.04%	2.77%	0.00%
**1b**	66.93%	2.85%	0.00%
**1c**	69.45%	0.01%	0.00%

The F∙∙∙HO intramolecular hydrogen bond has been found to be a dominating effect of the conformational isomerism in 2-fluorophenylboronic acid, and this can be related to a nonoperative n_F_→p_B_ interaction, that would be possible for an O–B–O moiety orthogonal to the phenyl ring. Also, an orthogonal O–B–O fragment relative to the ring would avoid the π_CC_→p_B_ interaction, which is highly stabilizing. The lack of any F/B interaction can be confirmed by the absence of a *J*_B,F_ coupling constant for the somewhat broad signal in the ^11^B NMR spectrum (Supporting Information File 1). In this way, it is not possible to mimic the n_F_→σ*_OH_ interaction in 2-fluorophenol by using the n_F_→p_B_ interaction in **1** to check for the existence of intramolecular hydrogen bonding in 2-fluorophenol. Thus, 2-substituted phenylboranes were used to evaluate the importance of the n_X_→p_B_ interaction for the rotational isomerism, since R = H experiences a much lower steric hindrance compared to OH and does not participate in hydrogen bonding.

**Figure 7:**
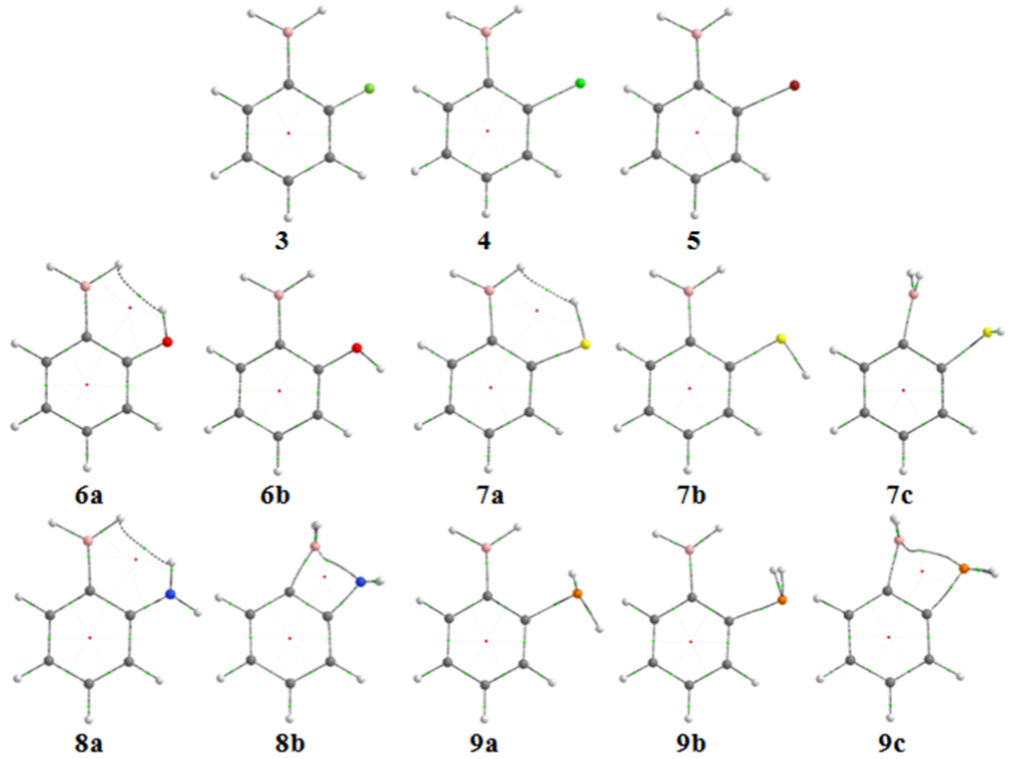
Molecular graphs indicating bond paths (BPs), bond critical points (BCPs; green dots), and ring critical points (RCPs; red dots) for the conformers of 2-substituted phenylboranes **3–9**.

Some F (**3**), Cl (**4**), Br (**5**), OH (**6**), SH (**7)**, NH_2_ (**8**) and PH_2_ (**9**) derivatives of phenylboranes were theoretically analyzed (Figure 7). Compounds **3–5** do not show any bond path between nonbonded atoms and, therefore, their conformation (H–B–H moiety coplanar to the aromatic ring) is governed by the strong π_CC_→p_B_ interaction (Table 5). As in the case of **1**, a hypothetical n_X_→p_B_ interaction for the halogen derivatives **3–5** is not sufficiently strong to stabilize the conformation with the H–B–H moiety orthogonal to the benzene ring, confirming the weak ability of halogens to participate in hydrogen bonds forming four- and five-membered rings. However, **6a**, **7a** and **8a**, the most stable conformers for the respective compounds, exhibit dihydrogen bonds, with electronic densities ρ(*r*) superior to that found for **1b**. Moreover, n_X_→π*_CC_ interactions are also highly stabilizing. Surprisingly, good electron donors, such as the nitrogen-containing phenylboranes, exhibit a conformation with the heteroatom lone pair directed toward the empty orbital of boron; this is the case for **7c**, **8b** and **9c**. Despite not being appreciably populated (high energy in Table 5) because of the loss in hyperconjugative energy due to the lack of π_CC_→p_B_ interaction, these geometries are at least located as local minima for **7**–**9**. The important hyperconjugation n_X_→p_B_ (especially for the NH_2_ derivative) obtained by NBO analysis (Table 5) indicates that N, S and P are considerably better electron donors than halogens in these cases. This interaction is explicitly expressed for **8b** and **9c** by means of X∙∙∙B bond paths in QTAIM (Figure 7). Indeed, the B–C–C(N) bond angle in **8c** and **9c** is significantly curved to allow the formation of a four-membered ring, reflecting the effectiveness of the X/B interaction.

**Table 5:** Conformational energies (in kcal mol^−1^) and hyperconjugative interactions (in kcal mol^−1^).

Parameter	**3**	**4**	**5**	**6a**	**6b**	**7a**	**7b**	**7c**	**8a**	**8b**	**9a**	**9b**	**9c**

*E*_rel_	–	–	–	0	3.1	0	0	5.1	0	9.1	0	1.5	5.7
n_X_→π*_CC_	–	–	–	32.7	30.0	19.1	18.0	–	39.2	–	3.0	–	–
π_CC_→p_B_	–	–	–	31.1	25.1	25.1	23.8	–	31.2	–	27.7	26.9	–
n_X_→p_B_	–	–	–	–	–	–	–	8.0	–	167.8	–	–	^a^
Total hyperconj.	6718.1	4246.9	17380.5	6398.4	6313.0	3416.5	3418.0	523.2	5867.8	752.1	4581.0	4622.6	517.3

^a^Does not appear because it is considered a covalent bond rather than a hyperconjugation, according to NBO.

## Conclusion

A parallelism between the empty p orbital of boron in 2-fluorophenylboronic acid and the σ*_OH_ orbital in 2-fluorophenol was evaluated to search for hydrogen bonding as the dominating interaction in the conformational equilibrium of these compounds, or otherwise. Indeed, in the case of 2-fluorophenol a hydrogen bond does not exist according to this approach, because the replacement of σ*_OH_ by a better electron acceptor (empty p orbital) to give the title compounds continues not to show any n_F_→p_B_ interaction, which is similar to the quantum version for the hydrogen bond n_F_→σ*_OH_. Despite the interference of a strong π_CC_→p_B_ interaction, it was shown that the n_F_→p_B_ interaction is not sufficiently strong to access an orthogonal orientation for the R–B–R (R = H and OH) moiety relative to the phenyl ring in the fluorine derivatives. This is corroborated by the absence of *J*_B,F_ coupling constant. Better electron donors than fluorine (N, P and S) perform such an interaction. An intramolecular hydrogen bond F∙∙∙HO appears in 2-fluorophenylboronic acid and it contributes for the conformational stability, since a six-membered ring is formed from this interaction, which is more efficient than an interaction giving a four- or five-membered ring.

## Experimental

2-Fluorophenylboronic acid was purchased from Sigma–Aldrich and used without further treatment. ^1^H and ^11^B NMR spectra were obtained from a Bruker Avance III 600 spectrometer operating at 600.2 MHz for ^1^H and 192.6 MHz for ^11^B, using ca. 2 mg mL^−1^ in benzene-*d*_6_ and 20 mg mL^−1^ in CD_3_CN solutions. The infrared spectrum was acquired in a BOMEM MB100 spectrometer from 0.1 M CDCl_3_ solution, using a liquid cell with NaCl windows and 0.5 mm spacer, collecting 32 scans at 1 cm^−1^ resolution. For the theoretical calculations, a Monte Carlo conformational search at the HF/6- 31G(d,p) level for compounds **1a**–**2c** was performed with the Spartan program [25]. For derivatives **3–9c**, the energy minima were identified by scanning the BCCX and HBCC(X) dihedral angles at the HF/6-31g++(d,p) level. Each minimum was subsequently optimized at the MP2/aug-cc-pVDZ (**1a–2c**) and B3LYP/aug-cc-pVDZ levels, followed by inspection of the harmonic frequencies, by using the Gaussian 09 program [26]. For **1a–2c**, the calculations were carried out both for the gas phase and implicit CH_3_CN solvent, by using the polarizable continuum model by Tomasi and co-workers (in its integral equation formalism [27]) and by using a cavity built up using the UFF (radii with spheres around each solute atom) at the same level of theory. Natural bond orbital (NBO) analysis [28] was carried out at the B3LYP/aug-cc-pVDZ level over the optimized geometries, as were QTAIM calculations by using the AIMAll program [29]. Finally, spin–spin coupling constant calculations were performed at the BHandH/EPR-III level in order to check for possible intramolecular hydrogen bonds through ^1^*^h^J*_F,H(O)_ and the n_F_→p_B_ interaction through ^1^*^TS^J*_F,B_ in 2-fluorophenylboronic acid.

## Supporting Information

File 1 ^1^H and ^11^B NMR spectra for 2-fluorophenylboronic acid. Potential energy surfaces for compounds **3–9**.

## References

[1] Yang W, Gao X, Wang B (2003). Med Res Rev.

[2] Chen X, Liang G, Whitmire D, Bowen J P (1998). J Phys Org Chem.

[3] Schinazi R F, Laster B H, Fairchild R G, Prusoff W H (1985). Antimicrob Agents.

[4] Schinazi R F, Prusoff W H (1985). J Org Chem.

[5] Lauer M, Wulff G (1983). J Organomet Chem.

[6] Wulff G (1982). Pure Appl Chem.

[7] Bhat K L, Howard N J, Rostami H, Lai J H, Bock C W (2005). THEOCHEM.

[8] Niedenzu K (1976). J Organomet Chem.

[9] Alkorta I, Elguero J, Denisov G S (2008). Magn Reson Chem.

[10] Cormanich R A, Moreira M A, Freitas M P, Ramalho T C, Anconi C P A, Rittner R, Contreras R H, Tormena C F (2011). Magn Reson Chem.

[11] Moreira M A, Cormanich R A, de Rezende F M P, Silla J M, Tormena C F, Rittner R, Ramalho T C, Freitas M P (2012). J Mol Struct.

[12] Silvi B, Kryachko E S, Tishchenko O, Fuster F, Nguyen M T (2002). Mol Phys.

[13] Dunitz J D, Taylor R (1997). Chem–Eur J.

[14] Takemura H, Ueda R, Iwanaga T (2009). J Fluorine Chem.

[15] Fonseca T A O, Freitas M P, Cormanich R A, Ramalho T C, Tormena C F, Rittner R (2012). Beilstein J Org Chem.

[16] de Rezende F M P, Moreira M A, Cormanich R A, Freitas M P (2012). Beilstein J Org Chem.

[17] Silla J M, Cormanich R A, Rittner R, Freitas M P (2013). J Phys Chem A.

[18] Cormanich R A, Freitas M P, Tormena C F, Rittner R (2012). RSC Adv.

[19] Weinhold F, Landis C R (2012). Discovering Chemistry with Natural Bond Orbitals.

[20] Bader R F W (1990). Atoms in Molecules: A Quantum Theory.

[21] Bader R F W (1991). Chem Rev.

[22] Bader R F W (1998). J Phys Chem A.

[23] Koch U, Popelier P L A (1995). J Phys Chem.

[24] Erdogdu Y, Güllüoğlu M T, Kurt M (2009). J Raman Spectrosc.

[25] (2000). Spartan Pro.

[26] (2009). Gaussian.

[27] Cancès E, Mennucci B (1998). J Math Chem.

[28] (2001). NBO.

[29] (2013). AIMAll.

