# Supplementary material for: Conformational analysis and intramolecular interactions in monosubstituted phenylboranes and phenylboronic acids
Source: Beilstein J Org Chem. 2013 Jun 11;9:1127–34. doi: 10.3762/bjoc.9.125 (PMC3701380; doi:10.3762/bjoc.9.125)
Supplement: File 1 — 1H and 11B NMR spectra for 2-fluorophenylboronic acid. Potential energy surfaces for compounds 3–9. [file Beilstein_J_Org_Chem-09-1127-s001.pdf]

Supporting Information

for

Conformational analysis and intramolecular interactions in monosubstituted phenylboranes and phenylboronic acids

Josué M. Silla[1], Rodrigo A. Cormanich[2], Roberto Rittner[2] and Matheus P. Freitas[1*§]

Address: [1]Department of Chemistry, Federal University of Lavras, P.O. Box 3037, 37200-000, Lavras, MG, Brazil and [2]Chemistry Institute, State University of Campinas, P.O. Box 6154, 13083-970, Campinas, SP, Brazil

Email: Matheus Puggina de Freitas[*] - matheus@dqi.ufla.br

[*]Corresponding author

[§]Tel.: +55 35 3829-1891; Fax: +55 35 3829-1271

^{1}H and ^{11}B NMR spectra for 2-fluorophenylboronic acid.

Potential energy surfaces for compounds **3–9**.

Table of Contents

Figure S1: ^{1}H NMR spectrum for **1** in C_6D_6 solution (2 mg mL^{-1})........ S2

Figure S2: ^{1}H NMR spectrum for **1** in CD_3CN solution (20 mg mL^{-1})........ S2

Figure S3: ^{11}B NMR spectrum for **1** in C_6D_6 solution (2 mg mL^{-1})........ S3

Figure S4: Potential energy surface for 2-fluorophenylborane........ S4

Figure S5: Potential energy surface for 2-chlorophenylborane........ S4

Figure S6: Potential energy surface for 2-bromophenylborane........ S4

Figure S7: Potential energy surface for 2-hydroxyphenylborane........ S5

Figure S8: Potential energy surface for 2-sulfanylphenylborane........ S5

Figure S9: Potential energy surface for 2-aminophenylborane........ S6

Figure S10: Potential energy surface for 2-phosphanylphenylborane........ S6

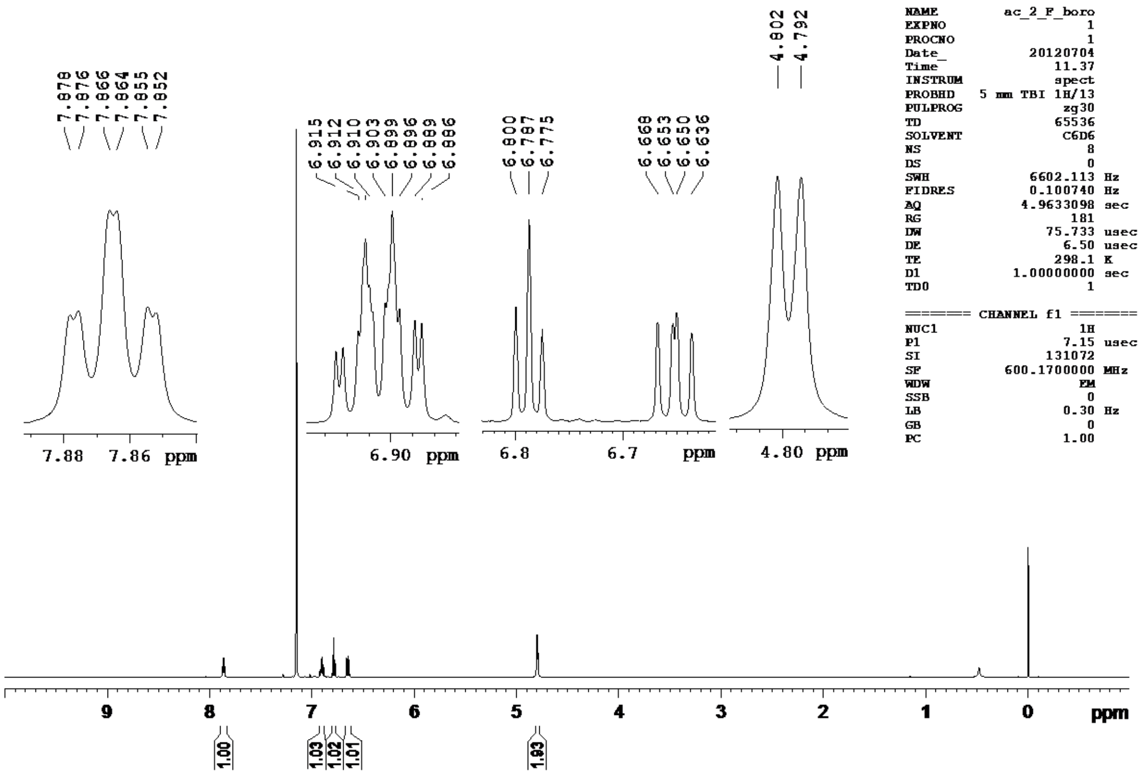

Figure S1: ^{1}H NMR spectrum for **1** in C_6D_6 solution (2 mg mL^{-1}).

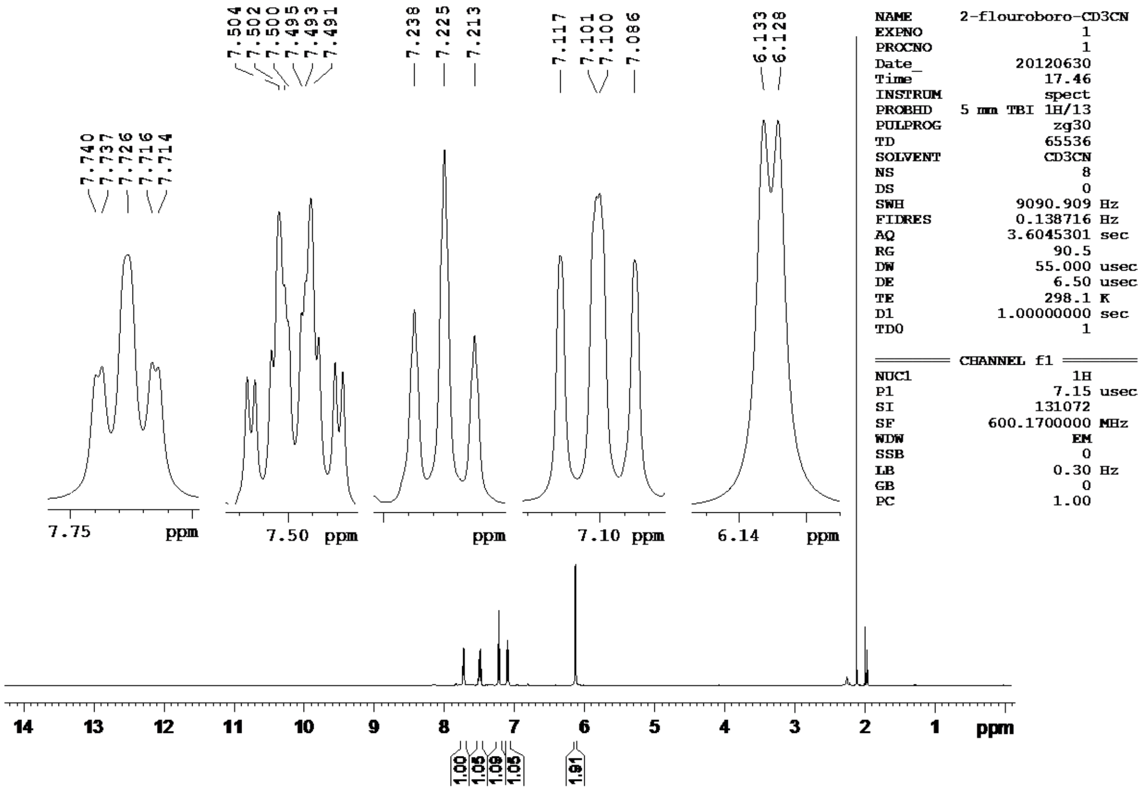

Figure S2: ^{1}H NMR spectrum for **1** in CD_3CN solution (20 mg mL^{-1}).

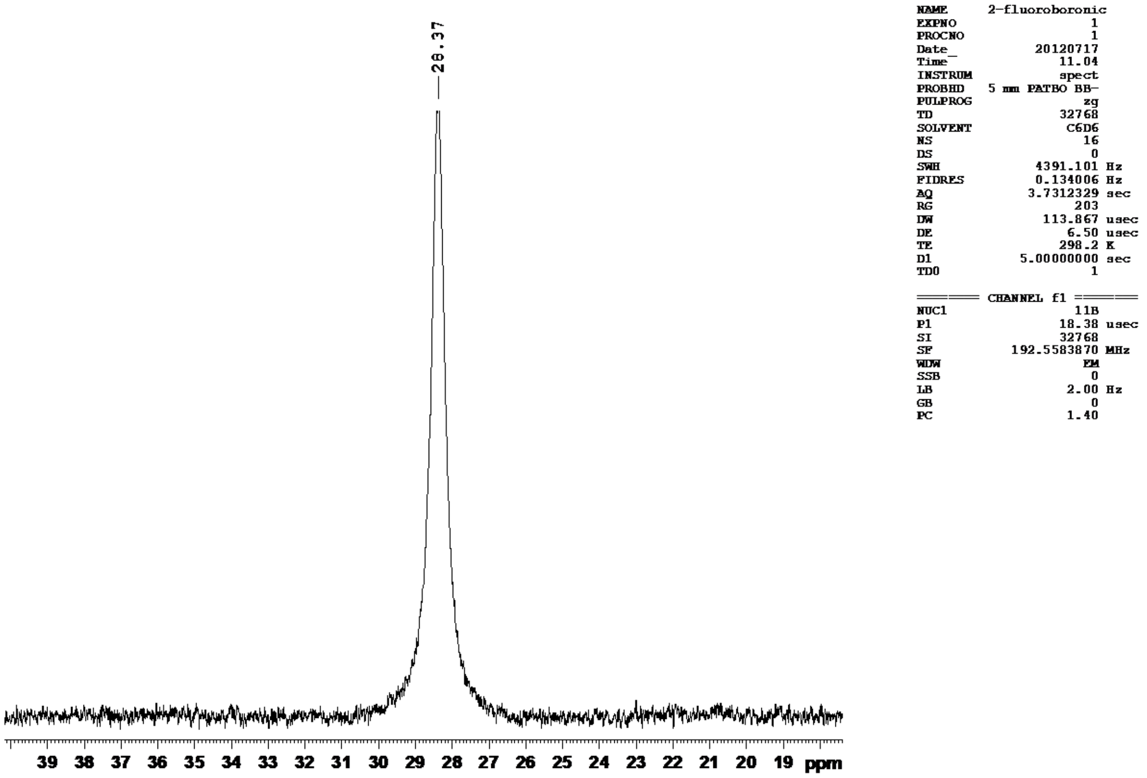

Figure S3: ^{11}B NMR spectrum for **1** in C_6D_6 solution (2 mg mL^{-1}).

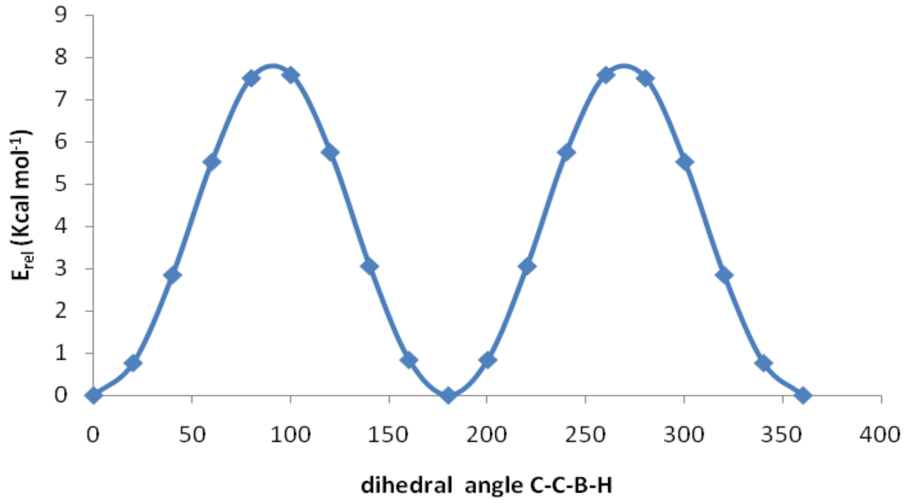

Figure S4: Potential energy surface for 2-fluorophenylborane **(3).**

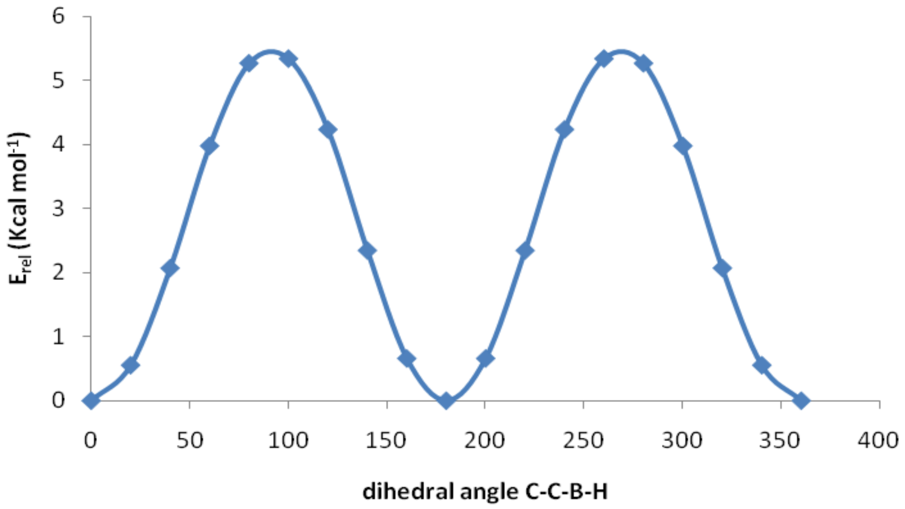

Figure S5: Potential energy surface for 2-chlorophenylborane **(4).**

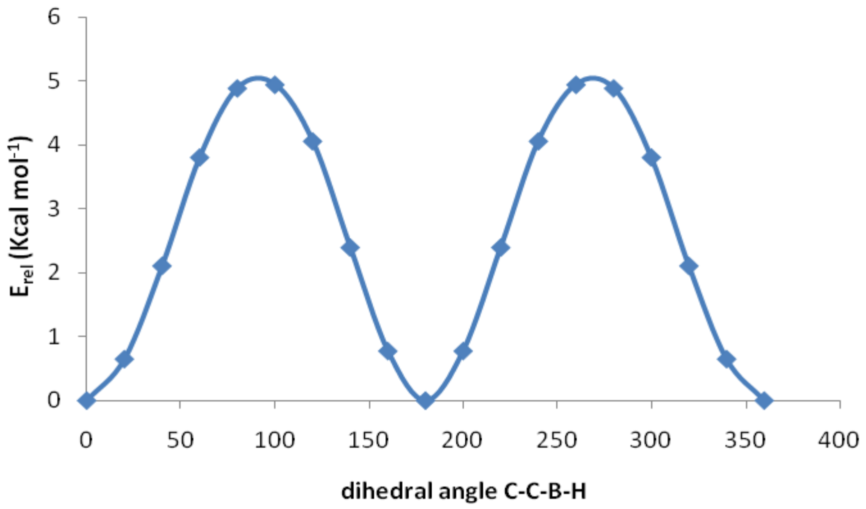

Figure S6: Potential energy surface for 2-bromophenylborane **(5).**

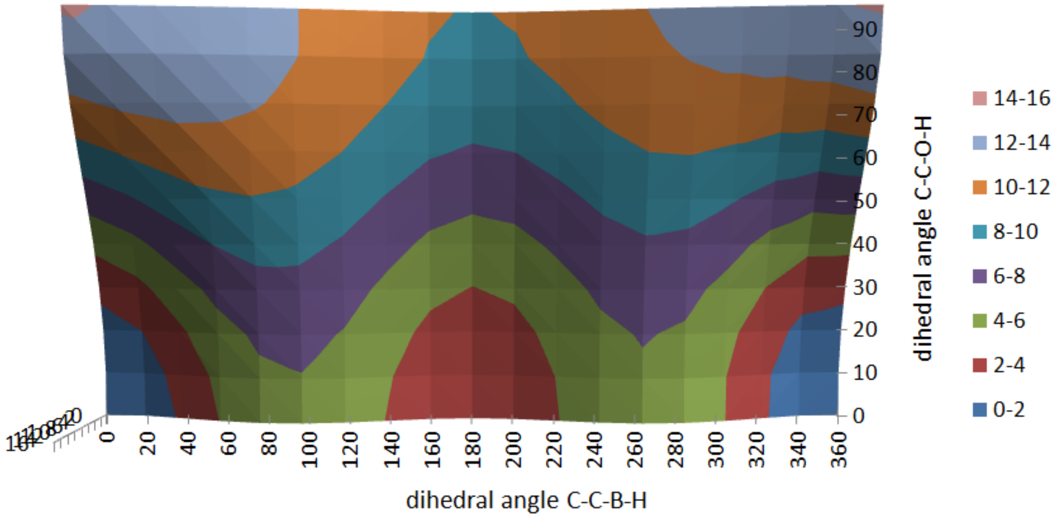

Figure S7: Potential energy surface for 2-hydroxyphenylborane **(6).**

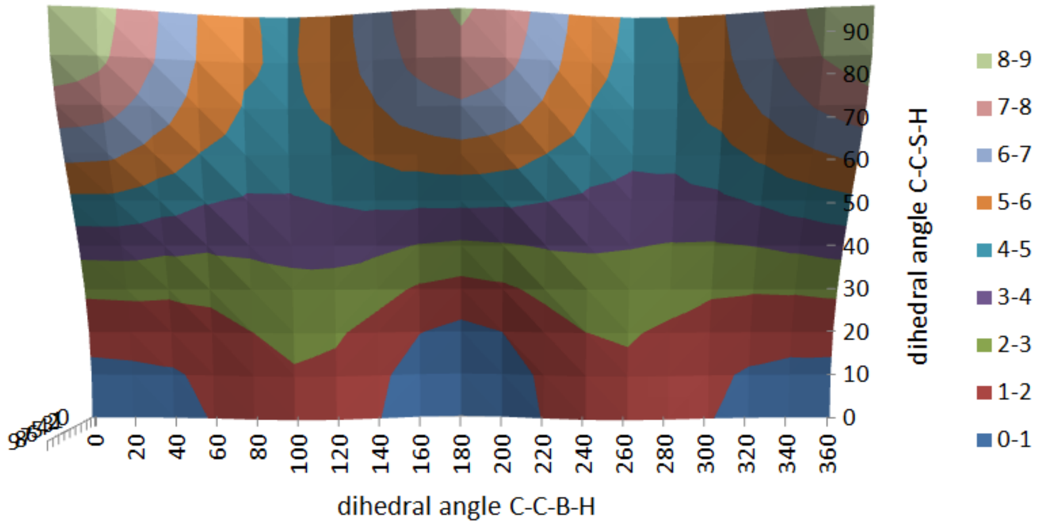

Figure S8: Potential energy surface for 2-sulfanylphenylborane **(7).**

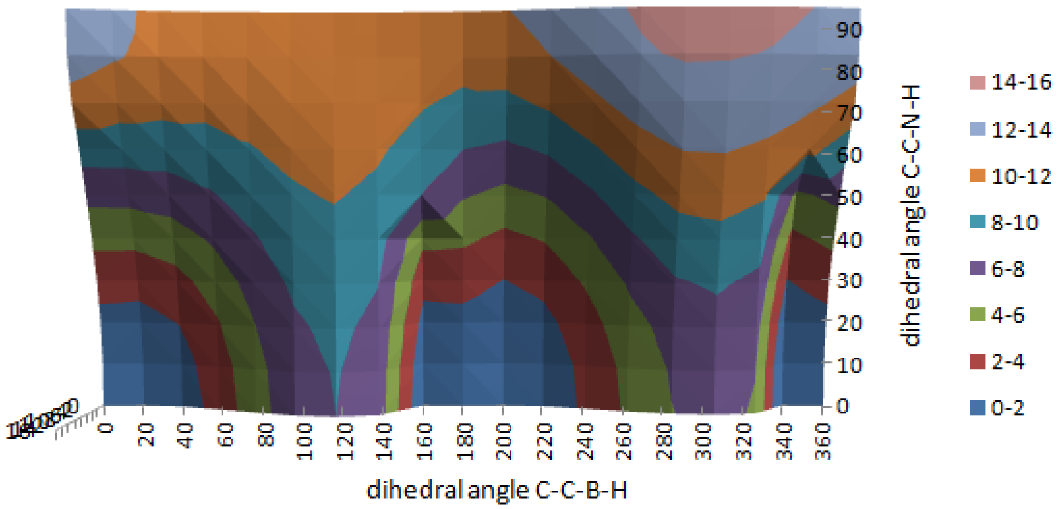

Figure S9: Potential energy surface for 2-aminophenylborane **(8).**

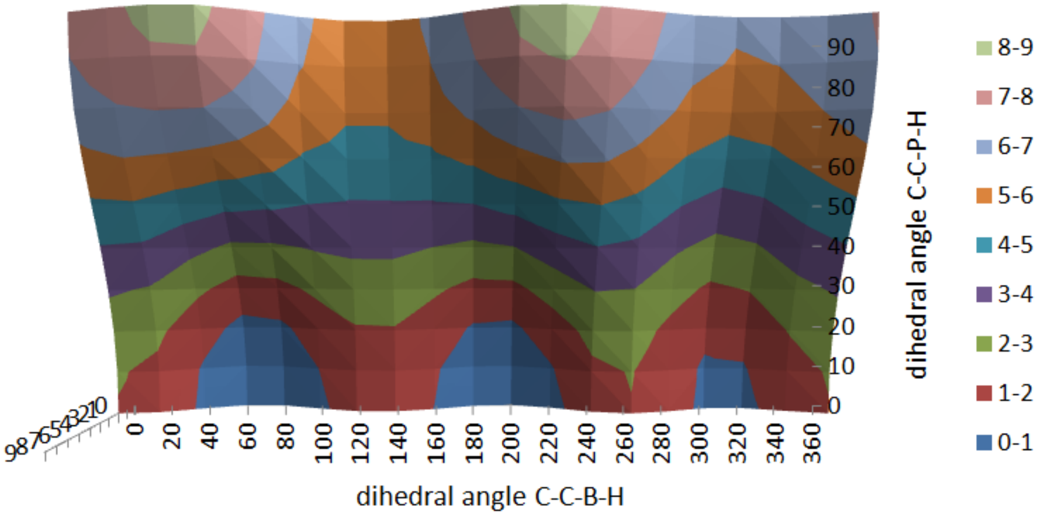

Figure S10: Potential energy surface for 2-phosphanylphenylborane **(9)**.
